# Obstetrical Society of London and Mr. Baker Brown

**Published:** 1867-10

**Authors:** 


### Obstetrical Society of London and Mr. Baker Brown.

The September *London Lancet* contains report of the special meeting for the purpose of considering and balloting upon the proposition brought forward by the Council at a previous meeting for the expulsion of Mr. Baker Brown. The whole discussion is full of shameful bitterness, professional jealousy, personal unkindness and all manner of uncharitableness. What reason for expulsion? If Mr. Baker Brown makes a surgical operation which is regarded as unjustifiable, say so, and publish your objections. If he thinks it is justifiable, let him say so, and publish his reason. Future generations of English surgeons must determine who is right—the present race are monomaniac, and will soon become inmates of madhouses, unless they cool down upon the subject of clitoridectomy. Mr. Baker Brown was voted out, but the real questions are as much unanswered as ever; nothing was gained, everything lost.

